# RNA Isoform Diversity in Human Neurodegenerative Diseases

**DOI:** 10.1523/ENEURO.0296-24.2024

**Published:** 2024-12-17

**Authors:** Christine S. Liu, Chris Park, Tony Ngo, Janani Saikumar, Carter R. Palmer, Anis Shahnaee, William J. Romanow, Jerold Chun

**Affiliations:** ^1^Sanford Burnham Prebys Medical Discovery Institute, La Jolla, California 92037; ^2^Biomedical Sciences Program, School of Medicine, University of California San Diego, La Jolla, California 92093

**Keywords:** Alzheimer’s disease, dementia with Lewy bodies, isoforms, long-read sequencing, Parkinson’s disease, RNA-seq

## Abstract

Single-nucleus RNA-sequencing (snRNA-seq) has revealed new levels of cellular organization and diversity within the human brain. However, full-length mRNA isoforms are not resolved in typical snRNA-seq analyses using short-read sequencing that cannot capture full-length transcripts. Here we combine standard 10x Genomics short-read snRNA-seq with targeted PacBio long-read snRNA-seq to examine isoforms of genes associated with neurological diseases at the single-cell level from prefrontal cortex samples of diseased and nondiseased human brain, assessing over 165,000 cells. Samples from 25 postmortem donors with Alzheimer's disease (AD), dementia with Lewy bodies (DLB), or Parkinson's disease (PD), along with age-matched controls, were compared. Analysis of the short-read libraries identified shared and distinct gene expression changes across the diseases. The same libraries were then assayed using enrichment probes to target 50 disease-related genes followed by long-read PacBio sequencing, enabling linkage between cell type and isoform expression. Vast mRNA isoform diversity was observed in all 50 targeted genes, even those that were not differentially expressed in the short-read data. We also developed an informatics method for detection of isoform structural differences in novel isoforms versus the reference annotation. These data expand available single-cell datasets of the human prefrontal cortical transcriptome with combined short- and long-read sequencing across AD, DLB, and PD, revealing increased mRNA isoform diversity that may contribute to disease features and could potentially represent therapeutic targets for neurodegenerative diseases.

## Significance Statement

Limited comparisons using single-cell transcriptomics analysis have been conducted among common neurodegenerative diseases. Here we identify new cell type and disease relationships involving known and novel mRNA isoforms by profiling single nuclei from human prefrontal cortices of Alzheimer's disease, Parkinson's disease, dementia with Lewy bodies, and nondiseased controls. Cell-type–specific RNA isoform diversity across the different diseases was examined using the combination of short-read snRNA-seq and targeted long-read single-nucleus isoform sequencing. We identified myriad novel transcripts that highlight an untapped understanding of RNA isoform diversity that exists within the brain and potentially contribute to human neurodegenerative diseases.

## Introduction

Neurodegenerative diseases cause a gradual loss of brain function and are estimated to affect over 50 million people worldwide, with projections that this number will only increase in the coming years (World Health Organization 2021; Alzeimer’s Association Releases, 2023). Broadly, all neurodegenerative diseases result in damage to and loss of cells in the nervous system. Some of these diseases, such as amyloidopathies, tauopathies, and synucleinopathies, are characterized by pathological accumulation of misfolded proteins. In Alzheimer’s disease (AD), an amyloidopathy and a secondary tauopathy, both amyloid-β plaques and neurofibrillary tangles composed of aggregated tau are present in the brain. Parkinson’s disease (PD) and dementia with Lewy bodies (DLB) are synucleinopathies, distinguished by ɑ-synuclein aggregates. These diseases can also be described by their clinical manifestations in addition to their pathological hallmarks. AD and DLB are both types of dementias, while PD is primarily a movement disorder. Despite their overlapping clinical manifestations and neuropathologies, it is unclear how these diseases compare and relate to each other on a single-cell transcriptional level (Smith et al., 2019; McAleese et al., 2021).

Prior studies have explored differences in the molecular complexity of neurodegenerative diseases using single-nucleus RNA-sequencing (snRNA-seq), which can delineate cell-type–specific gene expression changes within heterogeneous tissues like the brain (Luquez et al., 2022; Pozojevic and Spielmann 2023). Typical single-cell sequencing experiments employ short-read sequencing technology that effectively identifies differentially expressed genes (DEGs) but inherently limits the capture of full-length RNA isoforms. In AD, differential *APP* and *MAPT* isoform expression have been associated with plaque density and pathologies (Johnson et al., 1989, 1990; Moir et al., 1998; Bowles et al., 2022). Moreover, expression of genes involved in splicing and mRNA metabolic processes in the prefrontal cortex was found to be associated with AD pathology (Mathys et al., 2023), underscoring a need to understand the full range of expressed mRNA isoforms for a given gene.

Advances in long-read sequencing technologies have enabled comprehensive characterization of human brain RNA isoforms, including the discovery of novel isoforms (Leung et al., 2021; Palmer et al., 2021; Paoli-Iseppi et al., 2021). Combining long-read sequencing with single-cell or single-nucleus barcoding approaches can provide even higher resolution by revealing cell-type–specific RNA isoform expression (Gupta et al., 2018; Palmer et al., 2021; Tian et al., 2021; Hardwick et al., 2022).

Here, we report cell-type–specific RNA isoform diversity in the human prefrontal cortex from three neurodegenerative diseases—AD, DLB, and PD—along with nondiseased (ND) controls. Both shared and distinct transcriptomic changes on the gene level among three neurodegenerative diseases were observed, along with many novel transcripts not currently included in reference annotations and isoforms that showed cell-type–specific expression. Our data highlight the enormous diversity of RNA isoforms expressed within different cell types of the brain in health and disease, expanding upon the annotated transcriptome.

## Materials and Methods

### Human brain tissue

Control selection was based on the absence of neuropathology relevant to any of the three diseases and RNA integrity number (RIN) ≥ 6. Disease cases were chosen based on neuropathological confirmation of disease and RIN ≥ 6. Controls: 2 male, 3 female; AD: 4 male, 2 female; PD: 4 male, 3 female; DLB: 4 male, 3 female. Fresh frozen brain tissues from the prefrontal cortex (BA 8/9) were obtained from multiple brain banks (Extended Data Table 1-1) and stored at −80°C. Samples were moved briefly to a cryostat set at −20°C, and serial sections with all the cortical layers were obtained for nuclei isolation and RIN analysis. Efforts were made to remove white matter and enrich gray matter in the sections.

### RNA integrity number analysis

RNA was isolated from 20 µm sections using the RNeasy Mini kit (Qiagen #74106) and analyzed on an Agilent 4200 TapeStation using an Agilent High Sensitivity RNA ScreenTape assay.

### Single-nucleus isolation

Frozen 300 µm tissue sections were incubated in 1 ml of cold nuclei isolation buffer [320 mM sucrose, 100 µM EDTA, 1 µM DTT, 10 mM Tris-HCl pH 8, 5 mM CaCl_2_, 3 mM MgAc_2_, 0.1% Triton X-100, 80 U/ml RNase inhibitor (Takara Bio #2313A), cOmplete EDTA-free protease inhibitors (Roche #11836170001)] for 15 min and homogenized using a tissue grinder (Wheaton #358005) 10–15 times to mechanically dissociate the tissue. The homogenate was passed through a 50 µm filter to remove debris and centrifuged at 820 g for 5 min at 4°C. The isolated nuclei were washed twice with wash buffer (PBS with 1% EDTA, 250 µM EGTA, 1 µM DTT, 40 U/ml RNase inhibitor, protease inhibitors). Nuclei were stained with 1.25 µg/ml DAPI (Sigma #10236276001) diluted in the wash buffer for 15 min. The nuclei were sorted on a FACSAria Fusion (BD Biosciences) by gating out debris using the forward scatter and side scatter plots and selecting DAPI+ singlets to obtain ∼200,000 nuclei per sample. Sorted nuclei were diluted using PBS with 1% EDTA, 250 µM EGTA, 1,000 U/ml RiboLock RNase inhibitor (Thermo Fisher Scientific # EO0381) to a final concentration of ∼1,000 nuclei/ml using the Countess 2 Automated Cell Counter (Thermo Fisher Scientific #A27977).

### 10x Genomics single-nucleus 3′ cDNA library generation

The 10x Genomics Single cell 3′ v3.1 kit (protocol CG000204 Rev D with minor modifications) was used to encapsulate ∼10,000 nuclei from each sample into Gel beads-in-emulsions (GEMs). The standard protocol was used to generate barcoded, full-length cDNA libraries with reverse transcription time increased to 50 min and the extension time for cDNA amplification increased to 1.5 min; 25% of the cDNA library was fragmented to construct the final 3′ gene expression library. All QC steps were performed using the Fragment Analyzer (Advanced Analytical Technologies). The libraries were sequenced by Azenta Life Sciences on an Illumina NovaSeq 6000 at an average of 343 million reads per sample and 52,000 reads per cell.

### Unfragmented library reamplification using asymmetric PCR

Approximately 75% of the unfragmented library was used for target enrichment with a custom 50-gene probe panel (Twist Bioscience) and long-read sequencing. Genes of interest were selected based on their expression levels from our short-read snRNA-seq analysis, number of exons, and length of their transcripts for potential isoform discovery as well as evidence of involvement in neurological disorders based on literature. The libraries were first preamplified using a linear/asymmetric protocol adapted from Hardwick et al. (2022). For each sample, three independent reactions of 30 ng of cDNA were asymmetrically amplified using Platinum SuperFi II (Thermo Fisher Scientific #12368010) with the forward Partial Read 1 primer alone for 12 cycles. The PCR products were pooled and purified with 0.8× SPRISelect beads (Beckman Coulter B23318) and used as a template for the second, exponential PCR using forward Partial Read 1 primer and reverse Partial TSO primer for 4 cycles. Four independent reactions were run for each sample. The resulting PCR products were pooled and purified with 0.6× SPRIselect beads and eluted in 35 µl of elution buffer (Qiagen #19086). Partial Read 1 primer: CTACACGACGCTCTTCCGATCT; Partial TSO primer: AAGCAGTGGTATCAACGCAGAG; Thermocycler settings: 1 cycle of initial denaturation at 98°C for 30 s, 12 or 4 cycles of denaturation at 98°C for 10 s. annealing at 60°C for 10 s, extension at 72°C for 3 min and 30 s, and 1 cycle of final extension at 72°C for 5 min.

### Hybridization of libraries and target probes

The amplified cDNA libraries were enriched using a custom probe panel of 50 genes targeting exons in the coding sequence (CDS) region (Twist Bioscience) with the Standard Hybridization and Wash kit (Twist Bioscience #104178). First, the libraries were dried using a Vacufuge Plus (Eppendorf #022820168) at room temperature for an hour or until completely dehydrated. A 100 µM Oligo Pool (10 µl per sample) for blocking was made by mixing equal volumes of 200 µM Partial Read 1 primer and Partial TSO primer. Each dehydrated library was reconstituted in a Library Pool Solution comprising 7 µl of the Oligo Pool and 5 µl of 1 mg/ml Human Cot1 DNA (Thermo Fisher Scientific #15279011). Next, the Hybridization mix was heated at 65°C until all precipitate was dissolved. A Probe Solution was prepared for each sample by mixing 20 µl of the Hybridization mix, 4 µl of the custom Probe panel with 4 µl of nuclease-free water. The Probe Solution was heated at 95°C for 2 min and cooled on ice while the reconstituted Library Pool Solution was heated at 95°C for 5 min. Both the Probe and reconstituted Library Pool Solutions were brought to room temperature before being mixed thoroughly. Thirty microliters of Hybridization Enhancer were added on top of each reaction and then incubated at 70°C for 16 h to allow hybridization of the probes.

### Washes and Dynabead removal

Before the hybridization reaction was completed, 100 µl per sample of M-270 Dynabeads (Thermo Fisher Scientific #65305) was equilibrated to room temperature and washed thrice with 200 µl of Binding Buffer and resuspended in a further 200 µl of Binding Buffer. Once the hybridization reaction was completed, each reaction was rapidly transferred to an aliquot of washed M-270 Dynabeads and incubated at room temperature for 30 min on a Hula mixer (Labnet International #H5600). The reactions were then washed once with 200 µl Wash Buffer 1 at room temperature followed by three washes for 5 min at 48°C with Wash Buffer 2 (warmed to 48°C). The final wash was removed and 12 µl of nuclease-free water was added to form a DNA-Dynabead slurry. Separation of the DNA from the Dynabeads was achieved using Buffer DLB and Stop solution from the Qiagen REPLI-G kit (Qiagen #150023). Buffer D1 (sufficient for three samples) was prepared by mixing 9 µl of Buffer DLB with 32 µl of nuclease-free water, and Buffer N1 (sufficient for three samples) was prepared by mixing 12 µl of Stop Solution with 68 µl nuclease-free water. Twelve microliters of Buffer D1 were added to the DNA slurry and incubated at room temperature for 5 min. Twenty-four microliters of Buffer N1 were then added to the reaction, and the DNA was eluted from the beads by using a magnetic stand.

### Post-pulldown reamplification using exponential PCR and preparation of PacBio SMRTbell libraries

The enriched cDNA library was reamplified for 12 cycles with Platinum SuperFi II and Partial Read 1 and Partial TSO primers using the same PCR program as the pre-pulldown amplification. The amplified product was purified with 0.95× ProNex beads (Promega #NG2001). Ideally, 500 ng of cDNA was used to create PacBio HiFi barcoded SMRTbell libraries using the standard procedure (PacBio 101-892-000 Version 01) and the SMRTbell Express Template Prep Kit 2.0 (PacBio #101-685-400). Sequencing primer v4 was annealed to the libraries, and the Sequel II Binding kit 2.1 (PacBio #101-843-000) was used to bind the polymerase to the SMRTbell libraries. Each sample was sequenced on an individual SMRT Cell on the Pacbio Sequel II (8 M ZMWs) and generated an average of 5.1 million reads (Extended Data Table 1-1).

### Short-read sequencing data processing and filtering

10x Genomics Cell Ranger software (v4.0.0) was used to demultiplex samples, align reads to the reference genome, quantify unique molecular identifiers (UMIs), and create a cell-count matrix for every sample. Default parameters were used. A pre-mRNA reference file (ENSEMBL GRCh38) was supplied to capture intronic reads originating from unprocessed pre-mRNA species present in the nucleus. Using Seurat (v.4.3.0; Hao et al., 2021), sample matrices were filtered. Nuclei expressing fewer than 300 genes, containing >1% of reads mapping to mitochondrial RNA, and exceeding an outlier cutoff of UMIs determined by calculating the interquartile range were removed. Datasets were normalized using Seurat’s SCTransform() function with vst.flavor=”v2”.

### Integration, cell type identification, and differential expression analysis

SCTransform()-normalized datasets were integrated in Seurat using functions PrepSCTIntegration(), FindIntegrationAnchors(), and IntegrateData(). A previous dataset from human cortical samples (Lake et al., 2018) was used as a reference to label cell types in our samples using Seurat’s TransferData() function (dims = 1:30). The integrated data was scaled and UMAP embeddings were generated (dims = 1:50). Clusters were generated using FindNeighbors() (dims = 1:50) and FindClusters() (res = 0.3). Differentially expressed genes between various sample groups and cell types were identified using FindMarkers() using default parameters, where a gene is assigned as differentially expressed if: |log2fold| > 0.25, adj-*p* value < 0.05 according to a Wilcoxon *t*-test, with Bonferroni correction.

### Gene ontology overrepresentation analysis

Functional enrichment analysis for cell-type–specific DEGs for each disease relative to nondiseased samples was performed using the overrepresentation analysis module from WebGestaltR (v0.4.5; Wang et al., 2017). For analysis, a nonredundant set of gene ontology (GO) Biological Process terms was used as the GO database (Wang et al., 2017). GO terms with at least three annotated genes and a maximum of 300 genes were included. The background gene list (Wijesooriya et al., 2022) for each disease and nondiseased comparison was defined as genes that were expressed in at least two samples with a minimum average expression value of 0.001. Significant GO terms were those with a corrected false discovery rate of <0.05 using the Benjamini–Hochberg method.

### Long-read snRNA-seq data processing

Quality control and processing generally followed the recommendations in the cDNA_Cupcake repository (as of February 3, 2022). Subreads generated from the PacBio Sequel II were processed into HiFi reads using ccs (v6.2.0) with default parameters (–min-passes 3 –min-rq 0.99). Barcoded adapters and samples were demultiplexed using lima (v2.4.0). R1 and TSO 10x Genomics primer sequences were used to identify and remove reads with improper primer orientation using lima with the –isoseq parameter. isoseq3 (v3.4.0) tag was used to clip cellular barcode and UMI sequences and add them to the read metadata. Poly(A) tails were trimmed and concatemers were removed using isoseq3 refine. Reads were clustered and deduplicated based on cellular barcode and UMI to generate high-quality transcript clusters. Transcripts were mapped to the human reference genome (hg38) using minimap2 (v2.17-r941) and collapsed into nonredundant isoforms using cDNA_Cupcake’s collapse_isoforms_by_sam.py. The resulting unique isoforms were annotated with the GENCODE v39 reference annotation and filtered using a modified version of SQANTI3 (v4.1). cDNA_Cupcake’s link_molecule_to_celltype.py was used to match each SQANTI3-annotated isoform back to particular reads and their corresponding cellular barcodes and UMIs.

### Isoform comparisons

For each sample, SQANTI3-filtered classification files and genePred files, sample config files, experiment config files, and single-cell isoform files were used as inputs to isoSeQL (https://github.com/christine-liu/isoSeQL). The resulting database was loaded in python, and custom queries were used to generate plots and tables.

### Isoform functional domain annotation

orfipy (https://github.com/urmi-21/orfipy; Singh and Wurtele, 2021) was used to predict and select the longest coding ORF for each of the novel isoforms. The resulting fasta file was input into InterProScan (v5.70-102.0) (Paysan-Lafosse et al., 2023) to annotate these isoforms’ predicted functional domains.

## Results

### Cell-type annotation and differential gene expression analysis

**Figure 1.**
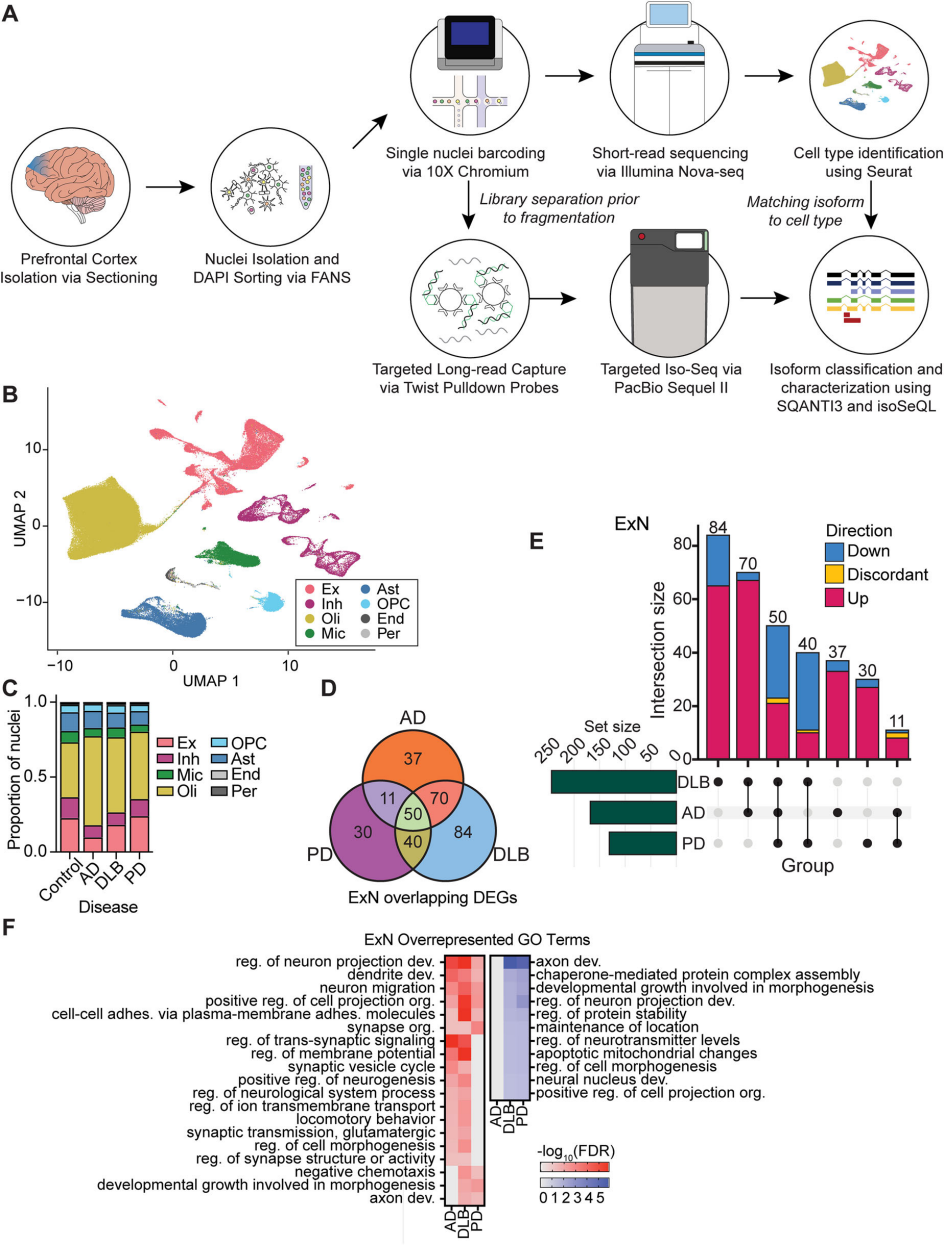
Transcriptomic profiling of excitatory neurons in AD, DLB, and PD using short-read snRNA-seq. ***A***, Experimental schematic of snRNA-seq and targeted Iso-Seq. ***B***, UMAP colored by cell type from AD, DLB, PD, and age-matched controls. ***C***, Proportion of cell types in each disease group summed across samples. ***D***, Venn diagram of DEGs in excitatory neurons from each disease group. ***E***, UpSet plot of excitatory neuron DEGs colored by directional overlap or discordance. ***F***, Heat map of overlapping GO pathways affected by each disease in excitatory neurons. Red is indicative of pathways involving upregulated genes and blue is indicative of pathways involving downregulated genes. Ex, excitatory neurons; Inh, inhibitory neurons; Mic, microglia; Oli, oligodendrocytes; OPC, oligodendrocyte precursor cells; Ast, astrocytes; End, endothelial cells; Per, pericytes. See Extended Data Figure 1-1 and Extended Data Tables 1-1, 1-2, and 1-3 for more details.

We profiled the transcriptome of the human prefrontal cortex from ND controls and three neurodegenerative diseases, AD, DLB, and PD, using targeted single-nucleus Iso-Seq (snIso-Seq). We performed short-read snRNA-seq and long-read isoform sequencing on the same barcoded cDNA libraries to enable matching isoforms from the long-read analysis back to cell types determined in short-read analysis (Fig. 1*A*). Similar to the SnISOr-Seq (single-nuclei isoform RNA-sequencing) approach (Hardwick et al., 2022), we reamplified the barcoded cDNA libraries using asymmetric PCR to selectively amplify barcoded cDNAs with poly(A) tails. Contrasting with SnISOr-Seq, which uses whole exome enrichment to remove only intron-containing sequences, we selectively enriched for transcripts of 50 disease-related genes by targeting probes to their exons in order to focus sequencing resources and obtain greater read depth to elucidate cell-type–specific isoform expression of these genes and capture rare isoforms. We selected 50 genes to target because previous snIso-Seq efforts have lacked sufficient sequencing depth across the entire transcriptome to enable comprehensive differential isoform expression analysis and to detect biologically relevant novel isoforms at the single-cell level, within the human brain (de la Fuente et al., 2020; Palmer et al., 2021).

10.1523/ENEURO.0296-24.2024.f1-1 Figure 1-1 Sample metadata and cell type proportions. (*A*) Plots showing the distribution of sex (left), age (middle), and RIN (right) of all samples. (*B*) UMAP plots colored by sex (left), RIN (middle), and age (right). (*C*) PCA plots colored by sex (left), RIN (middle), and age (right). (*D*) Cell type proportions in each individual sample. Download Figure 1-1, TIF file.

10.1523/ENEURO.0296-24.2024.t1-1 Table 1-1 **Sample information and sequencing QC.** Neuropathology and brain bank information are provided for each sample. Basic 10X QC and PacBio metrics for each sample demonstrate how many reads were obtained and various filters that were used. Download Table 1-1, XLS file.

10.1523/ENEURO.0296-24.2024.t1-2 Table 1-2 **Differential gene expression.** For each cell type and disease, comparisons were made to control samples. The resulting gene lists with adjust p-values are provided. Download Table 1-2, XLS file.

10.1523/ENEURO.0296-24.2024.t1-3 Table 1-3 **Gene Ontology Analysis.** Gene Ontology (GO) terms reported for each cell type’s differentially expressed genes in each of the diseases. Download Table 1-3, XLS file.

Samples for each group were age-, sex-, and RNA integrity number (RIN)-matched (Extended Data Fig. 1-1), and all samples had RIN ≥ 6. Overall, 25 brains (ND, *n* = 5; AD, *n* = 6; DLB, *n* = 7; PD, *n* = 7) were processed for short-read snRNA-seq (Extended Data Fig. 1-1, Extended Data Table 1-1). After quality control and filtering, 165,440 nuclei were profiled and clustered using Seurat (v4.3.0; Hao et al., 2021). Cell types were identified by projecting reference data onto our integrated Seurat object to transfer cell type labels to the appropriate clusters (Fig. 1*B*; Lake et al., 2018). Using the R package *propeller* to analyze differences in cell type proportions while accounting for covariates like sex, age, RIN, and disease (Phipson et al., 2022), we observed a statistically significant decrease in excitatory neurons in AD (Fig. 1*C*, Extended Data Fig. 1-1), while no other variables affected cell-type proportions. Furthermore, gene expression across the samples was not affected by sex, age, and RIN covariates as determined by principal component analysis (Extended Data Fig. 1-1).

We identified cell-type–specific DEGs (|log_2_fold| > 0.25, Bonferroni corrected *p* value < 0.05, Wilcoxon *t* test) across each disease relative to ND controls (Extended Data Table 1-2). For each disease, a different cell type appeared to be the most transcriptionally altered: 200 DEGs were identified in microglia in AD; 244 DEGs in excitatory neurons in DLB; and 170 DEGs in oligodendrocyte precursor cells (OPC) in PD (Extended Data Table 1-2). DLB shared more DEGs with AD than with PD across all the major cell types (Extended Data Table 1-2). In excitatory neurons, DLB shared 120 DEGs with AD and 90 DEGs with PD (Fig. 1*D*,*E*). This suggests that the neuronal molecular changes relative to ND observed in DLB are more similar to those observed in AD than PD. Gene ontology (GO) analysis was performed independently on the upregulated and downregulated DEGs from each cell type in each disease in order to identify overlapping biological processes among AD, DLB, and PD. Only the excitatory neuronal populations had multiple overlapping GO terms across the three diseases (Fig. 1*F*, Extended Data Table 1-3), involving neuron projection development and cell-to-cell adhesion. AD and DLB also shared GO terms involving synaptic signaling (Fig. 1*F*, Extended Data Table 1-3). PD and DLB had few overlapping affected GO pathways based on upregulated genes in excitatory neurons; however, several pathways overlapped between PD and DLB downregulated DEGs. One of these GO terms was “regulation of protein stability,” which included DEGs such as heat shock proteins HSP90AB1, HSP90AA1, and CLU (Extended Data Table 1-3). The downregulation of these heat shock proteins has been shown to affect ɑ-synuclein aggregation (Daturpalli et al., 2013; Lenzi et al., 2020). AD had no overrepresented GO terms with downregulated DEGs in excitatory neurons (Fig. 1*F*).

### Targeted long-read sequencing of single-nucleus cDNA libraries reveals isoform diversity

**Figure 2.**
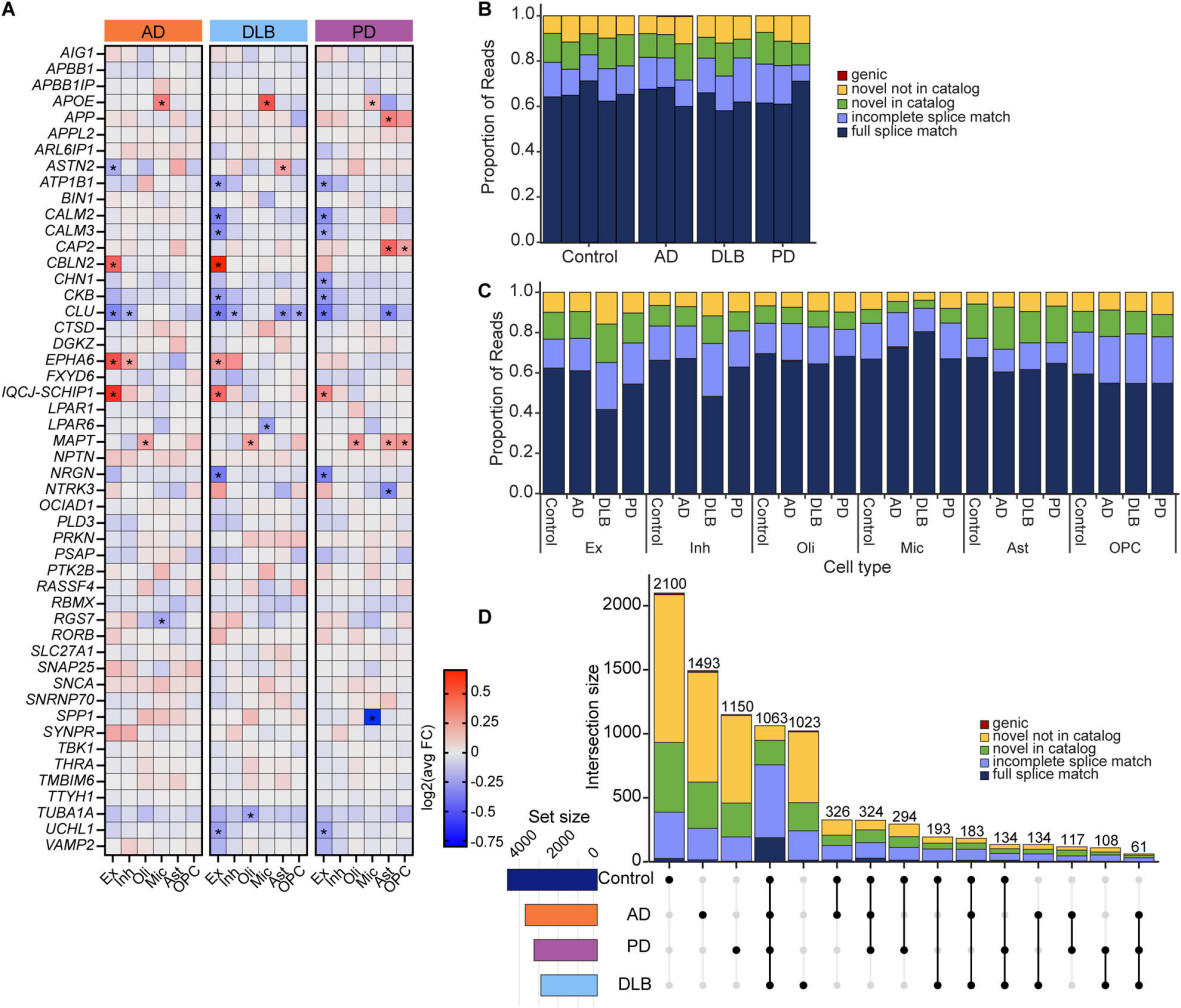
Isoform classification of 50 targeted genes in neurodegenerative diseases and control. ***A***, Heat map of gene expression changes of 50 targeted genes categorized by disease in the six major cell types. Asterisks (*) indicate significant DEGs (differentially expressed genes) in the specific cell type. ***B***, ***C***, Isoform structural classification and proportion of reads (***B***) in each individual, grouped by disease and (***C***) summed across individuals in each disease group by cell type. ***D***, UpSet plot of isoforms in different disease groups. Intersection and set size represent the number of isoforms. See Extended Data Table 2-1 for more details.

Previous reports using snIso-Seq demonstrated that sequencing a single sample on one Sequel II SMRT Cell was not sufficient to sequence the full expressed transcriptome and achieve sequencing saturation (Palmer et al., 2021). In an effort to achieve greater isoform coverage and sequencing depth, we therefore performed targeted snIso-Seq on 14 of the 25 cDNA libraries (*n* = 3 for each group except ND, *n* = 5; Fig. 1*A*; see Materials and Methods). Fifty genes with relevance to neurodegenerative diseases were selected for target enrichment based on the following criteria: relative expression in the brain based on our short-read snRNA-seq data (average expression > 0.05), average number of exons across known transcripts ≥3, average transcript length ≤4,000 bp, or evidence for involvement in neurodegenerative diseases (Blauwendraat et al., 2020; Orme et al., 2020; Leng et al., 2021; Bellenguez et al., 2022; Fig. 2*A*, Extended Data Table 2-1). Many genes, such as *BIN1*, *SNAP25*, and *SNCA*, were not differentially expressed in any major cell type; however, this does not preclude the possibility that different isoforms are being expressed without affecting the overall gene expression.

10.1523/ENEURO.0296-24.2024.t2-1 Table 2-1 **Target gene panel.** Various metrics associated with the fifty genes that were targeted for long-read sequencing and the number of isoforms and reads that were captured. Download Table 2-1, XLS file.

We obtained ∼107 million long reads from the 14 samples (∼3.8 to 6.3 million reads per sample; Extended Data Table 1-1). Reads were processed using isoseq3 and cDNA_Cupcake, and isoforms were annotated using a modified version of SQANTI3 (Tardaguila et al., 2018; Pardo-Palacios et al., 2024; described further below). The data were compiled for efficient comparison of transcripts across the different sample groups using isoSeQL (https://github.com/christine-liu/isoSeQL). The GENCODE v39 annotation was provided as an input to SQANTI3 to identify the different isoforms that were present and categorize them as full splice match (FSM) isoforms that match the reference; incomplete splice match (ISM) isoforms that are truncated relative to the reference; novel in catalog (NIC) isoforms that contain a novel combination of known splice sites; and novel not in catalog (NNC) isoforms that contain at least one novel splice site. In each of the samples, ∼60% of reads corresponded to FSM isoforms, and ∼20% of reads supported novel isoforms (NIC and NNC; Fig. 2*B*). No notable differences in the proportions of isoform categories were observed across individuals or diseases (Fig. 2*B*).

Long-read sequencing also captured the cell barcodes on each cDNA read, and thus, we could identify the cell type from which each read originated from the cell type assignment from short-read clustering (Fig. 1*B*). The proportions of FSM reads in excitatory and inhibitory neuronal populations were ∼40% in DLB and ∼60% in all other groups (Fig. 2*C*). Proportions of reads corresponding to novel isoforms in microglia were reduced across all diseases, possibly due to lower expression of some of the targeted genes in microglia and the smaller population of microglia in the brain relative to oligodendrocytes and neurons (Fig. 1*C*). Notably, a large number of novel isoforms were unique to a particular sample group (Fig. 2*D*). NNC isoforms tended to be supported by a small number of reads, and those with >50 reads were often detected in a single individual (Extended Data Table 3-1). On the other hand, some novel isoforms were shared across all sample groups (306 out of 1,063 isoforms in all sample groups; Fig. 2*D*) and were expressed in a majority of the samples. These results highlight the ability of long-read sequencing to discover isoforms that are not currently part of the reference annotation and are currently missed by standard short-read methods. We additionally observed that relatively few isoforms (61 isoforms) were shared by only the three neurodegenerative diseases but not the controls (Fig. 2*D*). These observations indicate that isoform diversity may contribute to different aspects of neurodegeneration; however, it is worth considering that only 50 genes were targeted and may not be representative of all genes and isoforms.

### Isoform heterogeneity and switching in full splice match reads

**Figure 3.**
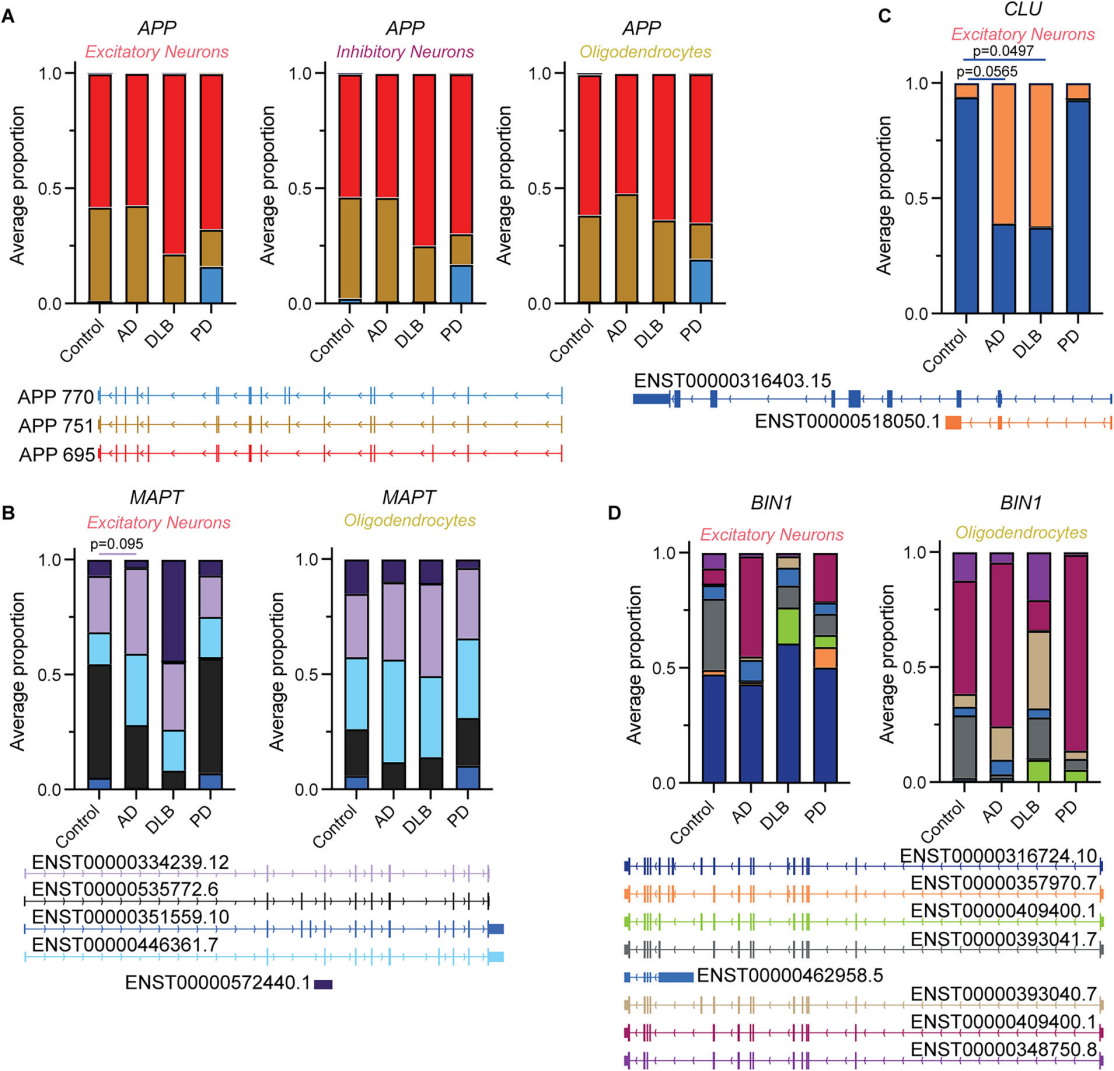
Proportion of FSM (full splice match) isoforms in *APP*, *CLU*, *BIN1*, and *MAPT* by cell type. Bar plot indicates average of sample read proportions for each isoform. Gene structure of the top expressed FSM isoforms is shown below. ***A***, *APP*; ***B***, *MAPT*; ***C***, *CLU*; ***D***, *BIN1.* For comparisons of isoform proportions between groups, the proportion of each gene isoform was calculated for a given sample and averaged within each group. Each isoform was compared to the control group, and one-way ANOVA, followed by Sidak's multiple-comparisons correction, was used to determine statistical significance. See Extended Data Figure 3-1 and Extended Data Tables 3-1 and 3-2 for more details.

Changes in the ratio of protein isoforms produced by the genes *APP* and *MAPT* have strong associations with AD and other tauopathies. For *APP*, mRNA isoform ratio changes between isoforms *APP-*751 (ENST00000357903) and *APP*-695 (ENST00000348990) have been reported in the AD cortex and hippocampus (Johnson et al., 1989, 1990; Moir et al., 1998). We examined these isoform changes in our data by calculating the proportion of FSM isoforms of each gene for a given sample and comparing them across disease groups and cell types. No change in the *APP*-751/695 ratio was observed between AD and ND controls in excitatory or inhibitory neurons (Fig. 3*A*, Extended Data Table 3-1). A small, insignificant increase was observed in oligodendrocytes. Oligodendrocytes have been shown to express the machinery necessary to produce amyloid-β protein (Gazestani et al., 2023), and this change in *APP*-751/695 ratio in AD could hint at their contribution to amyloid pathology. In DLB and PD, a decrease in the *APP*-751/695 ratio was observed in the neuronal populations

10.1523/ENEURO.0296-24.2024.f3-1 Figure 3-1 **Proportion of Full Splice Match isoforms.** Stacked bar plots of averaged FSM isoform proportion for each disease group and cell type. Each plot represents a gene from the target enrichment panel and only includes reads from isoforms categorized as FSM. NA represents reads from unassigned cell types. Download Figure 3-1, TIF file.

10.1523/ENEURO.0296-24.2024.t3-1 Table 3-1 **isoSeQL output.** Tabular output from isoSeQL listing the structural information for each isoform and the corresponding counts separated by cell type and sample. Download Table 3-1, XLS file.

10.1523/ENEURO.0296-24.2024.t3-2 Table 3-2 **InterProScan output.** Tabular output from InterProScan annotating the functional domains of protein sequences predicted from the isoform sequences detected through PacBio long-read sequencing. Download Table 3-2, XLS file.

We observed major isoform switching in *MAPT*, specifically in excitatory neurons from AD and DLB samples, contrasting with the lack of switching observed among *APP* isoforms (Fig. 3*A*,*B*). Major isoform switching is defined as a case in which the highest expressed isoform of a particular gene is different between two conditions. The major isoform in control samples, ENST00000535772.6 (black; which translates to the Tau 2N3R protein isoform), switched to ENST00000334239.12 (light purple; coding for the Tau 0N3R protein isoform) in AD and to ENST00000572440.1 (dark purple; a noncoding variant) in DLB. The observed increase in Tau 0N3R expression in AD (while not statistically significant; *p* = 0.095, one-way ANOVA, followed by Sidak’s multiple-comparisons correction) is consistent with another study where targeted MAPT long-read sequencing was performed (Bowles et al., 2022). Tau 0N3R is known to localize and enrich in neuronal axons, where its accumulation may lead to axonal degeneration (Bachmann et al., 2021). Additionally, ENST00000351559.10 (dark blue; Tau 2N4R protein isoform) was depleted in both AD and DLB relative to ND control. The *MAPT* isoform profile in excitatory neurons from PD looked indistinguishable from ND control, with no major isoform switching observed. In oligodendrocytes, no isoform switching was observed, and the major isoform, ENST0000446361.7 (light blue), was the highest expressed isoform across all disease groups (Fig. 3*B*).

Major isoform switching in excitatory neurons was also observed in other genes such as *CLU*, where control and PD expressed ENST00000316403.15 (blue) as the major isoform, while the major isoform in AD and DLB was ENST00000518050.1/ENST00000519472.5 (orange; Fig. 3*C*). Unlike *MAPT*, which was not a DEG in excitatory neurons in any of the three diseases according to the short-read snRNA-seq data, *CLU* was a downregulated gene (Fig. 2*A*), demonstrating that isoform changes can be independent of the gene expression changes between disease states. In fact, the major isoform in AD and DLB, ENST00000518050.1 (orange), is a noncoding variant, while the major isoform in control and PD, ENST00000316403.15 (blue), is the canonical *CLU* isoform. Our long-read isoform sequencing results indicate that there is a reduction in coding *CLU* isoform expression in AD and DLB (*p* = 0.0565 and *p* = 0.0495, respectively; one-way ANOVA, followed by Sidak’s multiple-comparisons correction), which would not have been detected using only short-read sequencing. Our results suggest that excitatory neurons may express noncoding *CLU* transcripts to regulate canonical *CLU* expression. We additionally examined the cell-type–specific isoform profile of *BIN1* in each disease group. We identified ENST00000316724.10 (dark blue) as the major isoform in excitatory neurons (Fig. 3*D*). However, each disease group expressed a different *BIN1* isoform pattern outside of the major isoform, with various other isoforms making up the rest of *BIN1* gene expression. In control, AD, and PD oligodendrocytes, ENST00000409400.1 (fuschia) was expressed as the major isoform, but in DLB, the major isoform switched to ENST00000393040.7 (beige; Fig. 3*D*). The wide variety in isoform expression was surprising, as *BIN1* was not differentially expressed in any cell types or disease groups (Fig. 2*A*). We observed similar major isoform switching events between cell types and diseases for other genes of interest in our targeted pulldown (Extended Data Fig. 3-1). The isoform heterogeneity we observed in known isoforms across the different cell types and diseases in the various genes is further compounded by the capture of novel isoforms and highlights the need to elucidate the roles specific isoforms can play.

### SQANTI3 modification annotates novel features of novel not in catalog isoforms

SQANTI3 categorizes isoforms relative to a user-supplied reference annotation into broad structural categories (Tardaguila et al., 2018; Pardo-Palacios et al., 2024). For the novel isoforms in particular, we sought to identify the structural features that make them unique. Novel in catalog (NIC) isoforms can result from a combination of known splice sites/junctions or the absence of a junction through full intron retention, and SQANTI3 annotates these subtypes accordingly. Novel not in catalog (NNC) isoforms have various features that differentiate them from the isoforms in the reference annotation. However, these NNC features are not identified or annotated by the current version of SQANTI3. Thus, we made fully integrated modifications to SQANTI3 in order to fully characterize these features as part of its implementation.

**Figure 4.**
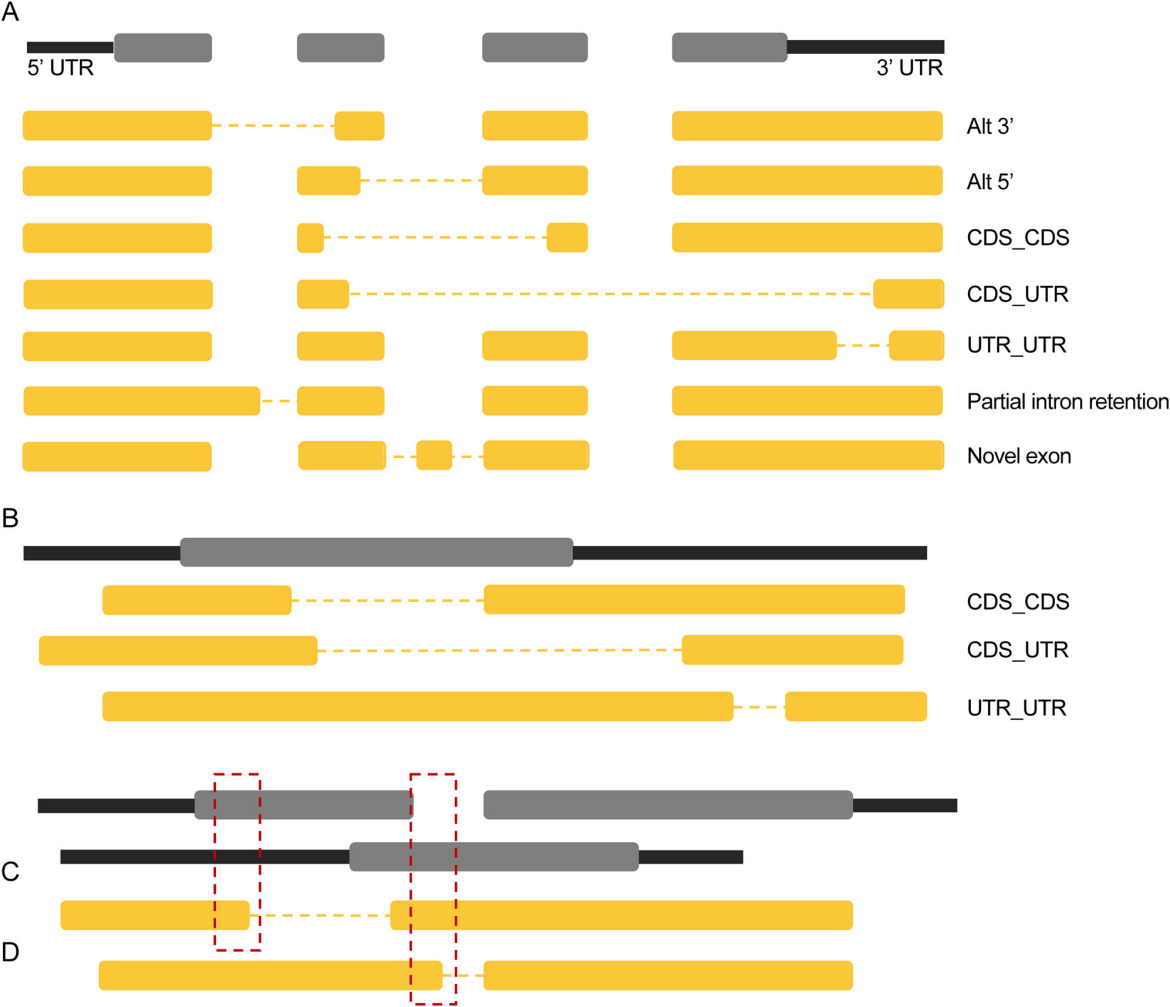
SQANTI3 categories and framework for annotating NNC (novel not in catalog) features. ***A***, Seven features of NNC isoforms that create a novel junction (or two). Multiple features can be observed in a single transcript, but only one is necessary to assign the isoform to the NNC category. Dotted line indicates the new junction. Black (UTR) and gray (CDS) bars represent the reference annotation with the spaces indicating introns. Yellow bars represent examples of NNC isoforms. ***B***, New categorization of spliced transcripts that map to a gene with a single exon. ***C***, Example gene with multiple reference isoforms and a novel isoform with a new splice site that occurs in the CDS of one reference isoform and UTR of the other reference isoform. ***D***, Novel isoform with a new splice site that occurs in the intron of one reference isoform of the gene and the CDS of the other reference isoform.

The modifications to SQANTI3 only involve the part of the tool that determines which category an isoform belongs to. Once an isoform is determined to diverge from the known set in the reference, its junctions and splice sites are examined more closely to determine whether it belongs in the NIC or NNC category. In our edited script, additional features of the NNC isoforms are identified and noted in the “subcategory” section of the classification output file. Each of these features on its own can make an isoform fit into the NNC category, or several can be present in a single isoform. These seven features are: Alt 3′ junction, Alt 5′ junction, CDS_CDS junction, CDS_UTR junction, UTR_UTR junction, partial intron retention, and novel exon (Fig. 4*A*). Alt 3′, Alt 5′, CDS_CDS, CDS_UTR, and UTR_UTR junctions all increase the size of junctions, and new splice sites are generated within known exons in the coding sequence (CDS) or untranslated regions (UTR), while partial intron retentions and novel exons decrease and split junctions, respectively, with new splice sites in annotated introns. Alt 3′ junctions result from a new acceptor splice site on the 3′ end of the junction that truncates the “acceptor” exon; the 5′ donor splice site is unchanged and matches a known splice site. Alt 5′ junctions follow the same pattern as Alt 3′ junctions, but the novel splice site is the 5′ donor. CDS_CDS junctions are created from two new splice sites within the coding sequence. These exons do not need to be adjacent, and both novel splice sites can occur in the same exon, splicing out coding sequence otherwise known as an exitron (Marquez et al., 2015). CDS_UTR junctions have two new splice sites within exons where one splice site is in the coding sequence and one splice site is in a UTR (on either end). Similarly to the CDS_CDS junction, the exons do not need to be adjacent, and the junction can occur within a single exon. UTR_UTR junctions have two novel splice sites that are both within a UTR; they can occur within the same UTR (5′ or 3′) or different ones (5′ and 3′). These three types of junctions that are made up of two novel splice sites within exons (CDS_CDS, CDS_UTR, and UTR_UTR) can also broadly be referred to as intra-exonic junctions (Lee et al., 2018). The extension of an exon into previously annotated intronic space by creation of a new splice site in intronic sequence describes partial intron retention. Novel exons create two new splice sites in intronic space by including sequence that does not overlap any known exon (Fig. 4*A*).

An additional minor modification was made with regard to how SQANTI3 handles single-exon genes. Originally, SQANTI3 would label an isoform with two exons that mapped to a single-exon gene, as intergenic. This splice pattern matches a CDS_CDS, CDS_UTR, or UTR_UTR junction, and modifications were made to the code to label these isoforms in this manner (Fig. 4*B*). For single-exon genes in particular, this CDS_CDS junction generates an exitron (Marquez et al., 2015) that removes internal regions of the protein and increases the protein diversity that can be generated from a single gene.

Many genes have more than one transcript, which can complicate the assignment of whether a novel splice site occurs in the CDS or UTR. While many transcripts often overlap exons and differ by which exons are present in combination, there are genes with vastly differing transcripts because of alternate start codons or sequences that can be spliced in or out in different isoforms. For genes with multiple isoforms that differ from each other substantially in terms of what is considered intronic versus exonic or CDS versus UTR in each isoform, we developed rules for describing novel features that involve those sequences instead of annotating them randomly. In the case where a novel splice site in a transcript overlaps a coordinate annotated as CDS in one transcript and UTR in the other, priority is given to labeling that site as being in CDS (Fig. 4*C*). Likewise, for a coordinate that is part of an exon in one isoform and part of an intron in another, priority is given to labeling that site as being exonic (CDS or UTR) (Fig. 4*D*).

### Novel isoforms account for the majority of expression in some genes

**Figure 5.**
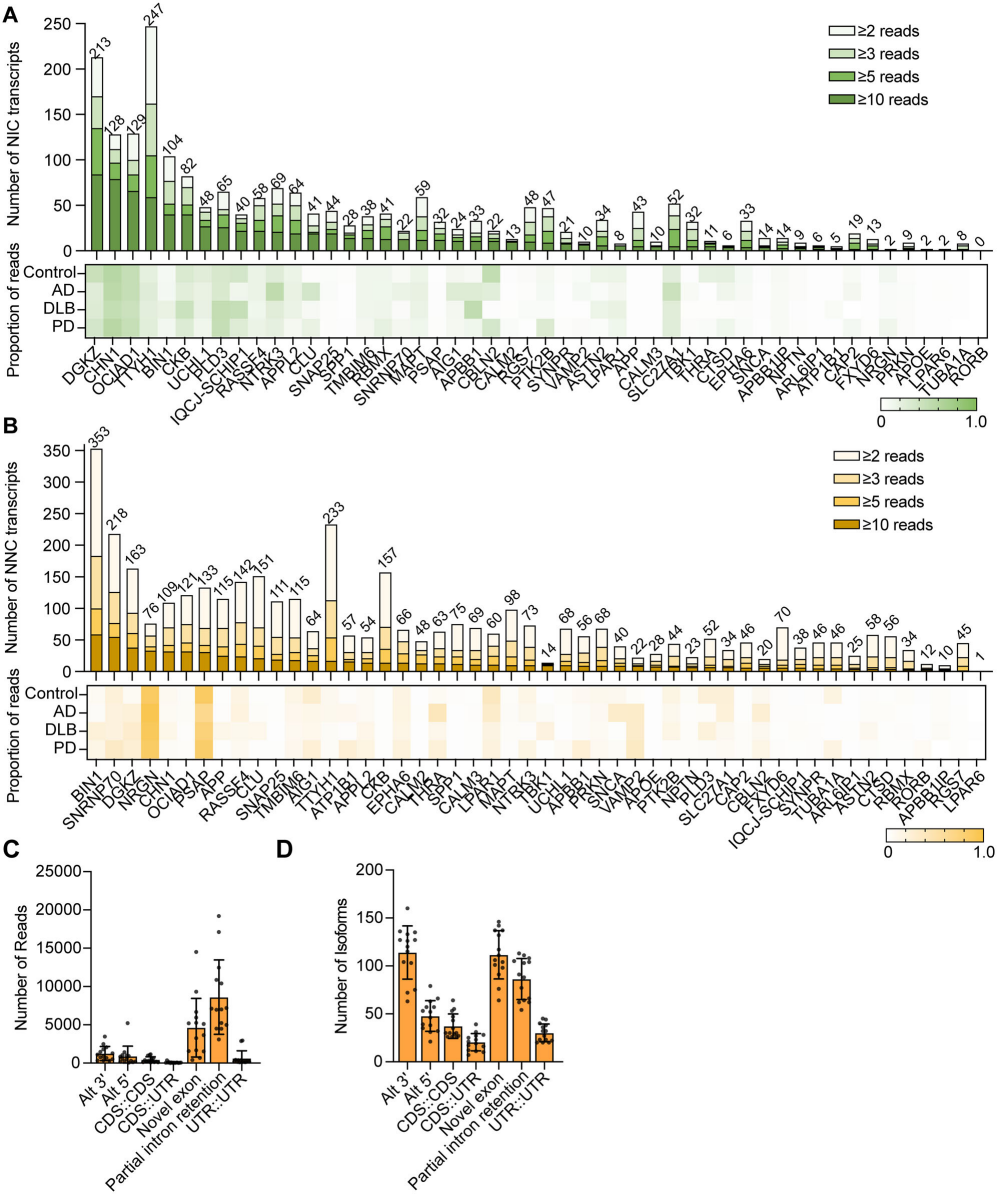
In-depth analysis of novel isoforms identified using targeted snIso-seq. ***A***, ***B***, Novel isoforms detected in each gene ranked by the number of unique transcripts (top). Proportion of novel isoforms [excluding ISM (incomplete splice match)] in each disease group represented in a heat map (bottom). Green represents (***A***) NIC (novel in catalog) isoforms and yellow represents (***B***) NNC (novel not in catalog isoforms). ***C***, ***D***, NNC features identified using modified SQANTI3 and quantified by (***C***) number of transcripts or (***D***) number of reads. Each black dot represents a sample. Statistical significance was assessed by one-way ANOVA, *p* < 0.0001. See Extended Data Figures 5-1, 5-2, and 5-3 for more details.

The novel isoforms we observed were further investigated by quantifying NIC and NNC isoforms by gene and calculating their relative levels of expression (Fig. 5*A*,*B*). *TTYH1*, *DGKZ*, *OCIAD1*, and *CHN1* were among the genes with the highest number of unique NIC isoforms with at least two reads (Fig. 5*A*), with these isoforms accounting for 20 to 58% of the gene expression as measured by read count proportion. The genes with the highest expression of NIC isoforms in each disease group were *CHN1* in control, PD, and AD (54–58%), and *APBB1* in DLB (50%; Fig. 5*A*, Extended Data Fig. 5-1). were readily FSM isoforms detected for both *CHN1* and *APBB1* across the majority of samples (Extended Data Fig. 5-1), suggesting that our targeted snIso-Seq approach captured a representative profile of these isoforms. *BIN1* had the greatest variety of NNC transcripts with a total of 353 unique NNC isoforms. Both *NRGN* and *PSAP* had high expression of NNC isoforms, ranging from 65 to 95% of the total read count in all of the sample groups (Fig. 5*B*). We did not observe a correlation between the number of transcripts or proportion of novel isoforms and the expected average transcript length or number of exons (Extended Data Fig. 5-2). Interestingly, several genes displayed a large proportion of ISM reads, including *ASTN2*, where very few reads from FSM transcripts were found in our data, suggesting that these transcripts may have been incompletely reverse-transcribed during the 10× library preparation (Extended Data Fig. 5-1, Extended Data Table 2-1) or that the enrichment method did not optimally capture them due to their length (most transcripts are >2,000 bp). Additionally, we were not able to examine differential expression of novel isoforms in disease as most novel isoforms had low read counts or were identified in a single sample (Extended Data Table 3-1).

10.1523/ENEURO.0296-24.2024.f5-1 Figure 5-1 **Structural category proportions of targeted genes.** Stacked bar plots showing the proportion of reads that support isoforms in each structural category for each gene from our enrichment panel. The number on top of each bar represents number of reads. Download Figure 5-1, TIF file.

10.1523/ENEURO.0296-24.2024.f5-2 Figure 5-2 **Novel isoform correlation with gene characteristics.** Relationship between average transcript length and number of NIC (top left) or NNC (top right) reads. Each dot corresponds to a gene in our enrichment panel. Relationship between average number of exons in isoforms of a gene and the number of NIC (bottom left) or NNC (bottom right) reads. Each dot represents a gene. Download Figure 5-2, TIF file.

10.1523/ENEURO.0296-24.2024.f5-3 Figure 5-3 **NNC features by disease group and cell type.** (*A*) Number of NNC isoforms with a particular NNC feature per sample grouped by disease. Bar represents the mean across samples, and error bars represent standard deviation. (*B*) Number of NNC isoforms with a particular NNC feature per sample grouped by cell type. Bar represents the mean and error bars indicate standard deviation. Download Figure 5-3, TIF file.

### Novel not in catalog features are not enriched in particular disease states

Using our modified version of SQANTI3, we examined which features differentiated the NNC isoforms from reference annotated isoforms. The feature most commonly observed in the NNC isoforms was partial intron retention, followed by novel exons (Fig. 5*C*). Partial intron retention differs from full intron retention by elongating an exon and creating a new splice site instead of creating one large exon by including the intron in between two exons. Intron retention may result from isolating RNA from nuclei and not cells, as splicing occurs in the nucleus before export to the cytoplasm. Incomplete mRNA processing could result in intronic retention, though most of these isoforms have other introns that were spliced out completely. As isoforms with noncanonical splice sites were removed from the analysis, the remaining, high-quality isoforms contain only canonical splice sites that could be recognized by the spliceosome. Many isoforms had Alt 3′ junctions, and a handful of isoforms had more than one novel junction (Fig. 5*D*). No significant difference was observed among diseases or cell types for each NNC feature, suggesting that the mechanism that produces these novel isoforms is not impacted by neurodegenerative disease or cellular identity (Extended Data Fig. 5-3).

### APP novel not in catalog transcripts in dementia

**Figure 6.**
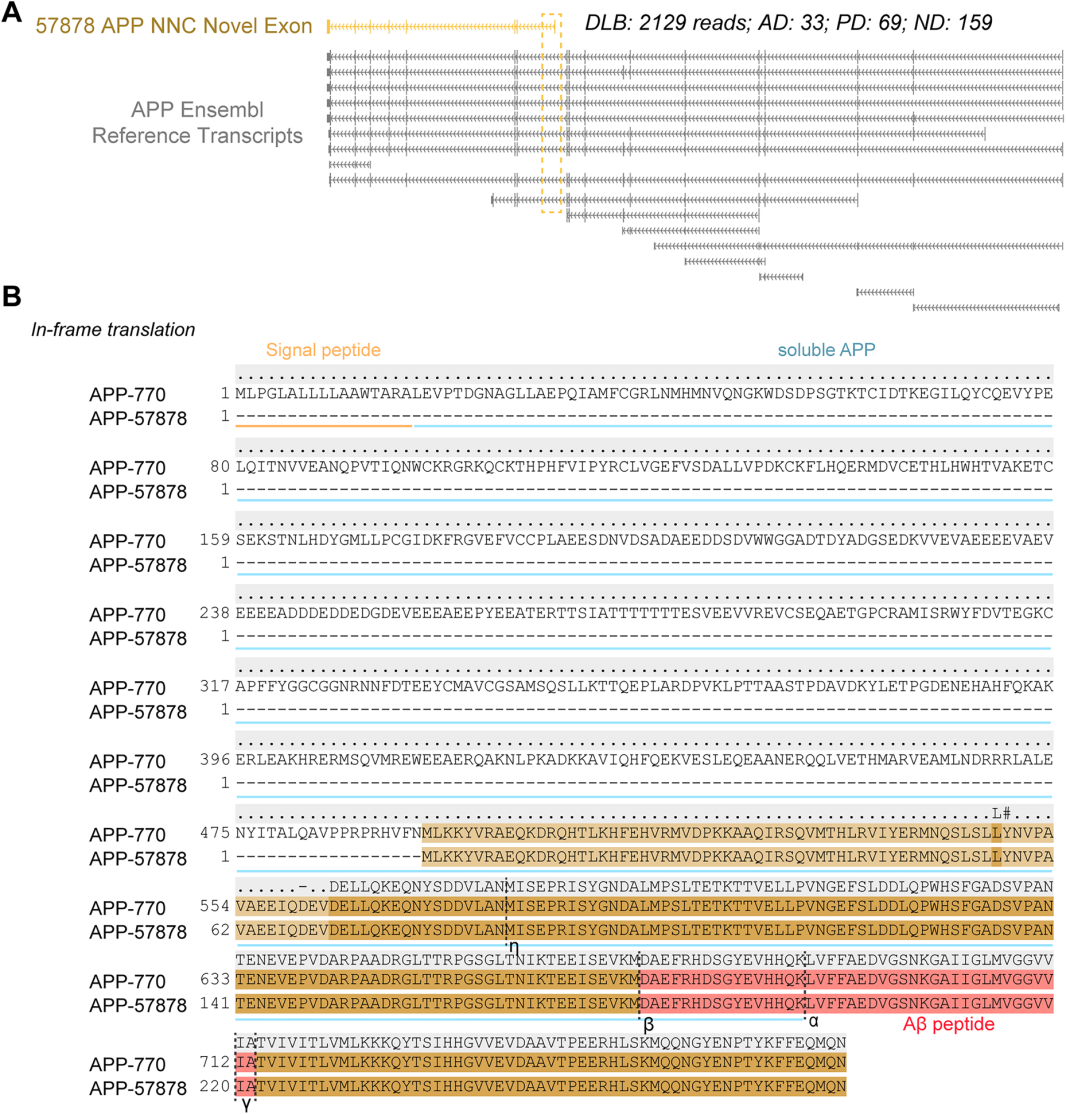
An example NNC (novel not in catalog) *APP* isoforms with a novel exon. ***A***, An *APP* NNC (novel not in catalog) isoform that is potentially translated into truncated protein isoforms lacking the soluble APP domain. ***B***, Alignment between the potential novel APP protein isoform with canonical full-length APP-770. Brown-shaded regions represent conserved sequences. Dotted vertical lines represent respective secretase cleavage sites. See Extended Data Figure 6-1 for more details.

While many novel isoforms were rare and only identified in a few reads in a small number of samples, several NNC isoforms were more prevalent. For example, an *APP* NNC isoform with novel exon was detected in all the sample groups and was highly expressed in DLB (Fig. 6*A*, Extended Data Table 3-1). *APP* NNC isoform 57878 was detected in 2,129 reads in DLB samples, compared to 159 reads in control, 33 in AD, and 69 in PD. It was detected in all three DLB samples; however, a majority of the reads (2,027 reads) were from a single sample (Extended Data Table 3-1); in this sample, reads were detected in all cell types, with increased read counts in both excitatory and inhibitory neurons.

10.1523/ENEURO.0296-24.2024.f6-1 Figure 6-1 **Select examples of NNC transcripts.** The NNC transcript is shown in reference to Ensembl reference transcripts. In-frame translations of these NNC transcripts are aligned with the canonical protein isoform. Domains of full-length canonical proteins are highlighted to demonstrate loss of functional domains in the theoretically translated diseased isoform. Download Figure 6-1, TIF file.

*APP* isoform 57878 was in-frame and could be translated into an N-terminally truncated APP protein isoform with all of the major cleavage sites of the various secretases intact, though missing the signal peptide that promotes surface expression (Fig. 6*B*). The absence of the N terminus may prevent the generation of soluble APP that inhibits β-secretase (Obregon et al., 2012; Peters-Libeu et al., 2015; Willem et al., 2015), and therefore the samples expressing these NNC isoforms may potentially lack a protective mechanism limiting amyloid-β plaque formation, which could contribute to the progression of DLB. While this isoform was more common than many of the others, identifying the same isoforms in additional samples by increasing sample number or by achieving sequencing saturation in our current samples along with functional studies are necessary to more confidently link them to disease. Other potentially interesting NNC isoforms were seen specifically in disease samples, where functional protein domains are missing (Extended Data Fig. 6-1). For each of the novel isoforms (including novel in catalog and novel not in catalog isoforms), we used InterProScan (v5.70-102.0) to annotate predicted functional domains (Extended Data Table 3-2). For example, many novel *APP* isoforms retained the amyloid-β peptide domain (IPR013803) while lacking or showing insignificant associations with the E1 cysteine rich N-terminus E1 domain (IPR008154), indicating N-terminal truncation. These data demonstrate the ability of our targeted snIso-Seq method to detect novel isoforms in a cell-type–specific manner with potential biological implications.

## Discussion

To our knowledge, this study represents the first combined snRNA-seq and targeted snIso-Seq comparison among AD, DLB, PD, and controls. The short-read snRNA-seq analysis identified DEGs in each disease relative to healthy controls and was combined with single-nucleus long-read sequencing to identify cell-type–specific isoforms in these diseases that would otherwise not be detected with only short-read sequencing. Our targeted, single-cell, long-read sequencing method allowed us to examine 50 genes’ isoforms in greater depth than would have been possible through untargeted, whole transcriptome, single-cell, long-read sequencing, and we identified novel isoforms in multiple genes. These novel isoforms were characterized in detail using SQANTI3, which we modified to annotate the features that made them distinct from the reference.

Our short-read differential expression analysis allowed us to look for transcriptomic changes that were unique to a particular disease or were shared between two or more diseases. While AD, DLB, and PD have some overlapping neuropathologies and clinical manifestations, previous studies have not examined whether these diseases also share transcriptional alterations as well. Genes that had altered expression between disease and control in each cell type were identified. While DLB and PD both exhibit Lewy body aggregation, DLB actually shared more DEGs with AD than with PD across all major cell types. The contribution of disease comorbidities may also be present, as previously reported based upon neuropathology but could also reflect shared mechanisms that deserve further study (Brenowitz et al., 2017a,b,c). GO analyses further indicated that many biological processes affected by changes in gene expression were unique to disease and cell type, with only excitatory neurons in all three diseases sharing multiple GO terms.

The targeted snIso-seq approach helped to address the lower throughput from using PacBio long-read technology. While using long reads has advantages in capturing full-length isoform structures, the number of sequencing reads obtained is limited in comparison to short-read technologies. Additional sequencing could be performed to increase the number of reads, but this is limited by cost as well as sample material. Using a targeted gene enrichment method allowed us to better capture the diversity of expressed isoforms by increasing the sequencing depth on specific genes. We assessed 50 genes that were expressed in the brain and had been previously and independently linked to one or more of AD, DLB, and PD. While multiple genes were not differentially expressed in our short-read snRNA-seq data, we subsequently observed isoform heterogeneity and switching in multiple genes and cell types. These results highlight how snIso-Seq can be used to detect cell-type–specific isoform changes.

Indeed, for all 50 targeted genes, we identified novel isoforms, with some novel isoforms contributing substantially to overall gene expression. Expanding on the broad structural categories provided by SQANTI3, we made modifications that add annotation information about novel junctions or splice sites present in NNC isoforms. Seven features of NNC isoforms were defined, and our modified version of SQANTI3 was able to detect these changes in several isoforms. No particular novel isoform features were enriched in any disease state, which potentially indicates that disease-causing transcriptomic changes are not the result of global splicing changes that create specific types of novel features. Of note, we were able to identify isoforms with novel splice junctions within the CDS or UTR exons, otherwise known as intra-exonic junctions (IEJs), which could result from the expression of somatically recombined genes in the brain and/or through novel splice activities (Lee et al., 2018; Palmer et al., 2021). These isoforms along with all the other novel isoforms not only demonstrate the power of long-read sequencing to identify novel gene forms that had been previously overlooked but also expand the catalog of predicted proteins that can be generated from the human genome. Predicted protein coding potential and functions of novel isoforms suggested that several novel isoforms were missing functional domains indicating that if these isoforms are translated, they may function differently or aberrantly. Identifying these novel isoforms in disease states may provide insight into how they contribute to disease.

In this study, we focused on characterizing tissue samples from the prefrontal cortex (Brodmann areas 8 and 9). The prefrontal cortex may not be representative of all affected regions of the brain in the neurodegenerative diseases studied, all of which have particular regions that are specifically impacted, like the substantia nigra in PD and the entorhinal cortex in AD. We limited our long-read sequencing analysis to only 50 genes to obtain greater sequencing depth on their isoforms, which could not be obtained using unbiased approaches. For genes *NRGN* and *PSAP*, we identified predominantly NIC/NNC transcripts, most with intronic retention features, which may be due to the extraction of nuclear RNA from postmortem tissue, similarly observed in SnISOr-Seq (Hardwick et al., 2022). Many isoforms were only observed in individual samples, highlighting the diversity of isoform expression that is consistent with somatic genomic mosaicism present in the normal and diseased brain (Rohrback et al., 2018; Kaeser and Chun 2020; Costantino et al., 2021). Higher throughput methods to increase the number of interrogated cells and number of sequencing reads should emerge in the future to better capture rare isoforms. We do note that while this study represents the largest sample size to date for a snIso-Seq study, the sequencing resources required for sufficient sequencing depth of all categories of isoforms are prohibitive. Kinnex/MAS-seq single-cell sequencing that concatenates multiple cDNAs into longer fragment libraries (Al'Khafaji et al., 2023) or depletion methods using CRISPR may be advantageous in future studies to provide additional sequencing depth.

In summary, we developed a bioinformatics method for detailed annotation of novel isoform structures and observed enormous RNA isoform diversity expressed in various cell types of the human brain across three different neurodegenerative diseases and healthy controls, underscoring a need to characterize the full range of transcripts present in each cell. Our new tool and these datasets should be useful for further analyses of isoforms in the normal and diseased human brain, toward identifying disease-related isoforms and comprehensively annotating novel isoform structures. Known and novel RNA isoforms are undoubtedly relevant to neurodegenerative disease mechanisms and deserve further study, including their potential for identifying new biomarkers and therapeutics for neurodegenerative diseases.

## Data Availability

Isoform tracks colored by isoform structure and split by sample are available at https://genome.ucsc.edu/s/csl022/20230228_smallBrain

Modified SQANTI3: https://github.com/christine-liu/SQANTI3/tree/SQANTICL

isoSeQL: https://github.com/christine-liu/isoSeQL

All raw and processed sequencing data generated in this study have been submitted to the European Genome-Phenome Archive (EGA) under accession code EGAS50000000132. For privacy reasons, these data are access controlled.

## Synthesis

Reviewing Editor: Deanna Smith, University of South Carolina Decisions are customarily a result of the Reviewing Editor and the peer reviewers coming together and discussing their recommendations until a consensus is reached. When revisions are invited, a fact-based synthesis statement explaining their decision and outlining what is needed to prepare a revision will be listed below. The following reviewer(s) agreed to reveal their identity: NONE. Note: If this manuscript was transferred from JNeurosci and a decision was made to accept the manuscript without peer review, a brief statement to this effect will instead be what is listed below.

In their manuscript "RNA isoform diversity in human neurodegenerative diseases" Liu et al., reports single nucleus sequencing from nuclei isolated from prefrontal cortices from Alzheimer's disease, dementia with Lewy bodies, and Parkinson's disease compared to age and sex matched controls without pathology. snRNA-seq analysis has been done before (Luquez 2022 ;Pozojevic 2023), but there is novelty in identification of different mRNA isoforms in the diseased frontal cortex vs. controls. These findings could potentially lead to significant discoveries in future experiments. As the authors mention the sequencing data for the 50 targeted genes chosen to examine isoform differences in transcripts will serve as a resource to the field of neurodegeneration, which is important. However, follow up experiments to the sequencing results (even if only qPCR) would strengthen the manuscript and validity of the data, especially the novel isoforms that are specifically expressed in disease states.

There are some issues that need to be addressed to strengthen the manuscript.

Specific comments from Reviewer 1:

1. It's exciting that transcripts that were not detected as DEG showed isoform differences and disease-specific expression. The discussion of these findings and verification by PCR would increase the novelty and importance of this paper.

2. There is a concern that statistics were not reported (considering there were at least 3 samples per group for DEG and major isoform switching. Significance was noted in the text without an accompanying p value.

3. Abbreviations are over used in the text making it hard to read. I would suggest spelling out as many abbreviations as possible.

4. Please clarify if the analysis of the APP isoforms was from a single sample. It's not clear why this in depth analysis of APP was included if it was from a single sample (Figure 6).

5. The text on line 513 the text states "other potentially interesting NNC isoforms were seen in disease samples where functional protein domains are missing (Fig. 6-1, table 3-1). Table 3-1 is labeled Table 5 and does not contain any information on proteins and their functional domains. This is really a very interesting point that should be included and discussed in the main text as these proteins will be different between disease states.

Overall, the manuscript provides a lot of sequencing information without a lot of discussion on the biological implications. The discussion should be expanded with a focus on the novel findings.

Specific comment from reviewer 2:

1. The link between the the short- and long-read sequencing experiments is not clear given the selection criteria for choosing the 50 genes for long-read sequencing. As written, the manuscript describes two loosely-related RNA sequencing projects.

2. The targeted selection of 50 candidates for long-read sequencing is very likely to miss novel genes of interest that may be involved in disease pathogenesis. Can this be better justified?

3. The significance of isoform diversity is unclear. For example, in Fig. 3, no differences are observed with APP. For the differences seen with genes like MAPT, what impact do the observed isoforms have on translation? In order to conclude that these findings are biologically significant, it is important that the authors follow up on a couple specific isoforms that are significantly different. Without this the relationship between isoforms and disease state is tenuous.

Minor concerns:

1. In the introduction, the authors should provide some additional justification for choosing the pre-frontal cortex for analysis in RNA-seq experiments.

2. Define SnISOr-Seq when first introduced (line 256). Since the authors are making a direct comparison to the technique, it would be useful to briefly describe the approach.

3. In Fig. 1C, a key for the abbreviations used needs to be provided.

4. The significance of shared DEGs in excitatory neurons between groups needs to be discussed.

5. An effort should be made to focus on biology. The manuscript is difficult to read for someone not familiar with transcriptomics. The technical language could be better written or better explained for a general neuroscientist reader.
