# Supplementary figures and images for: RNA Isoform Diversity in Human Neurodegenerative Diseases
Source: eNeuro. 2024 Dec 17;11(12):ENEURO.0296-24.2024. doi: 10.1523/ENEURO.0296-24.2024 (PMC11693435; doi:10.1523/ENEURO.0296-24.2024)

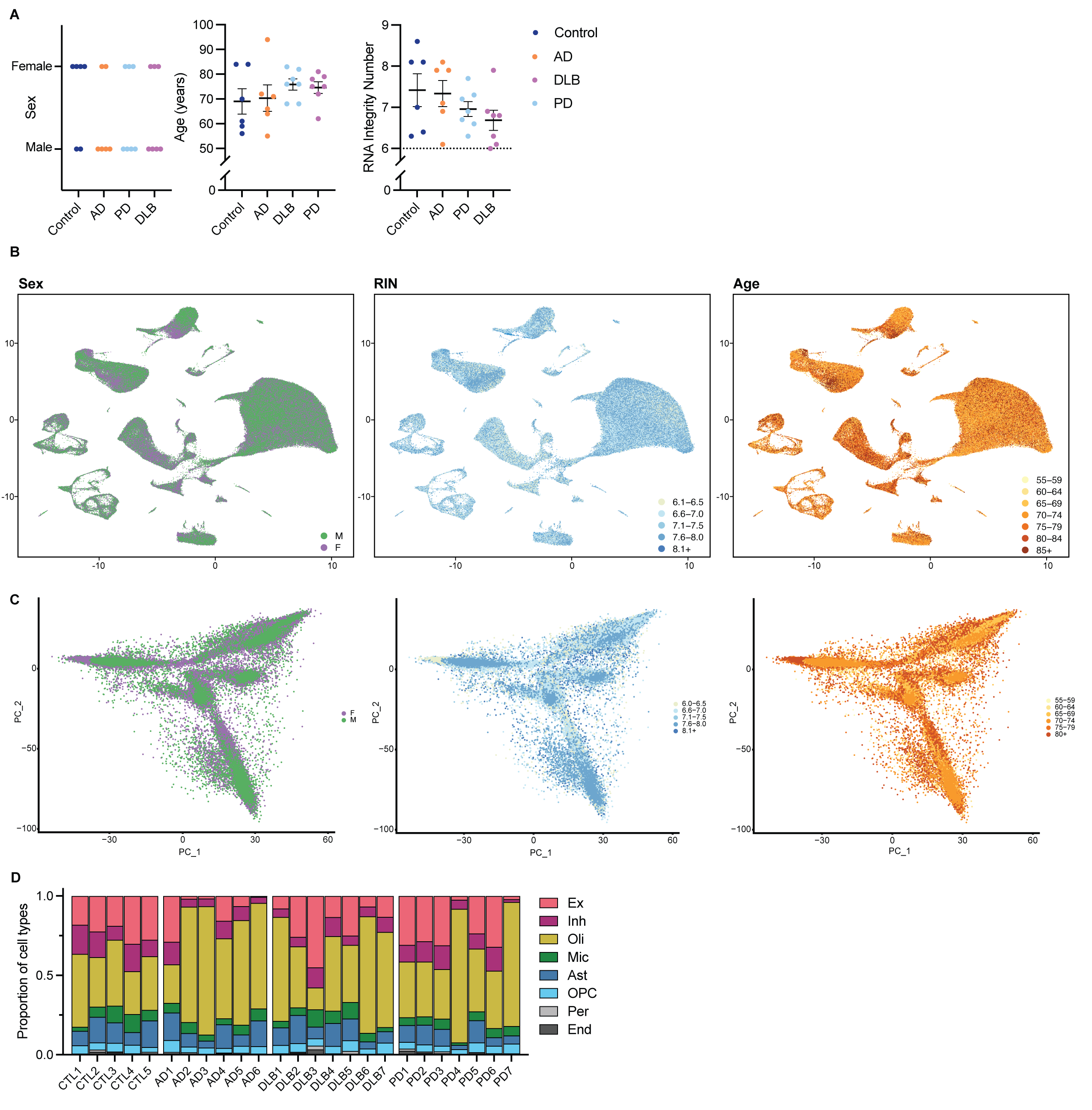

Supplement: Figure 1-1 — Sample metadata and cell type proportions. (A) Plots showing the distribution of sex (left), age (middle), and RIN (right) of all samples. (B) UMAP plots colored by sex (left), RIN (middle), and age (right). (C) PCA plots colored by sex (left), RIN (middle), and age (right). (D) Cell type proportions in each individual sample. Download Figure 1-1, TIF file. [file eneuro-11-ENEURO.0296-24.2024-s007.tif]

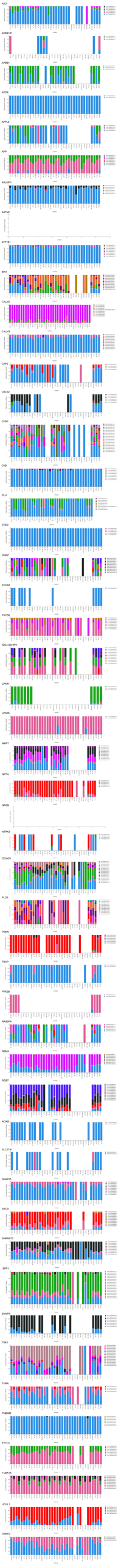

Supplement: Figure 3-1 — Proportion of Full Splice Match isoforms. Stacked bar plots of averaged FSM isoform proportion for each disease group and cell type. Each plot represents a gene from the target enrichment panel and only includes reads from isoforms categorized as FSM. NA represents reads from unassigned cell types. Download Figure 3-1, TIF file. [file eneuro-11-ENEURO.0296-24.2024-s008.tif]

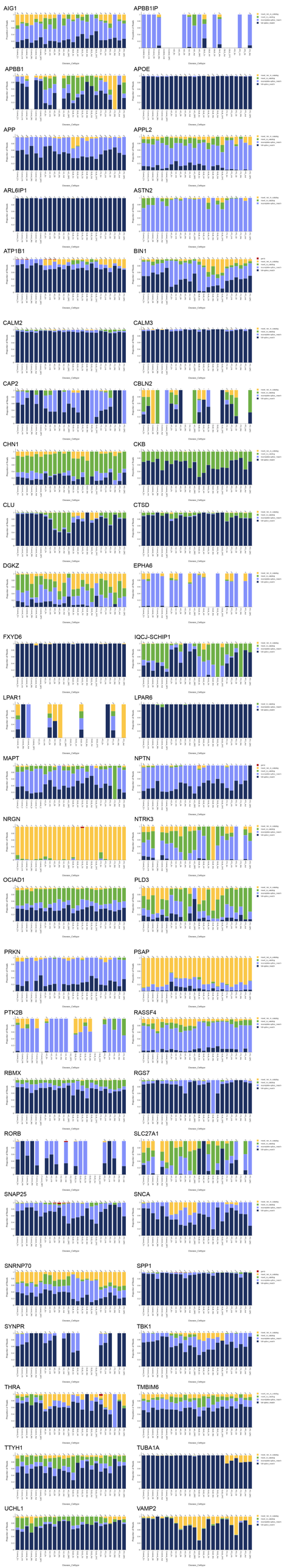

Supplement: Figure 5-1 — Structural category proportions of targeted genes. Stacked bar plots showing the proportion of reads that support isoforms in each structural category for each gene from our enrichment panel. The number on top of each bar represents number of reads. Download Figure 5-1, TIF file. [file eneuro-11-ENEURO.0296-24.2024-s009.tif]

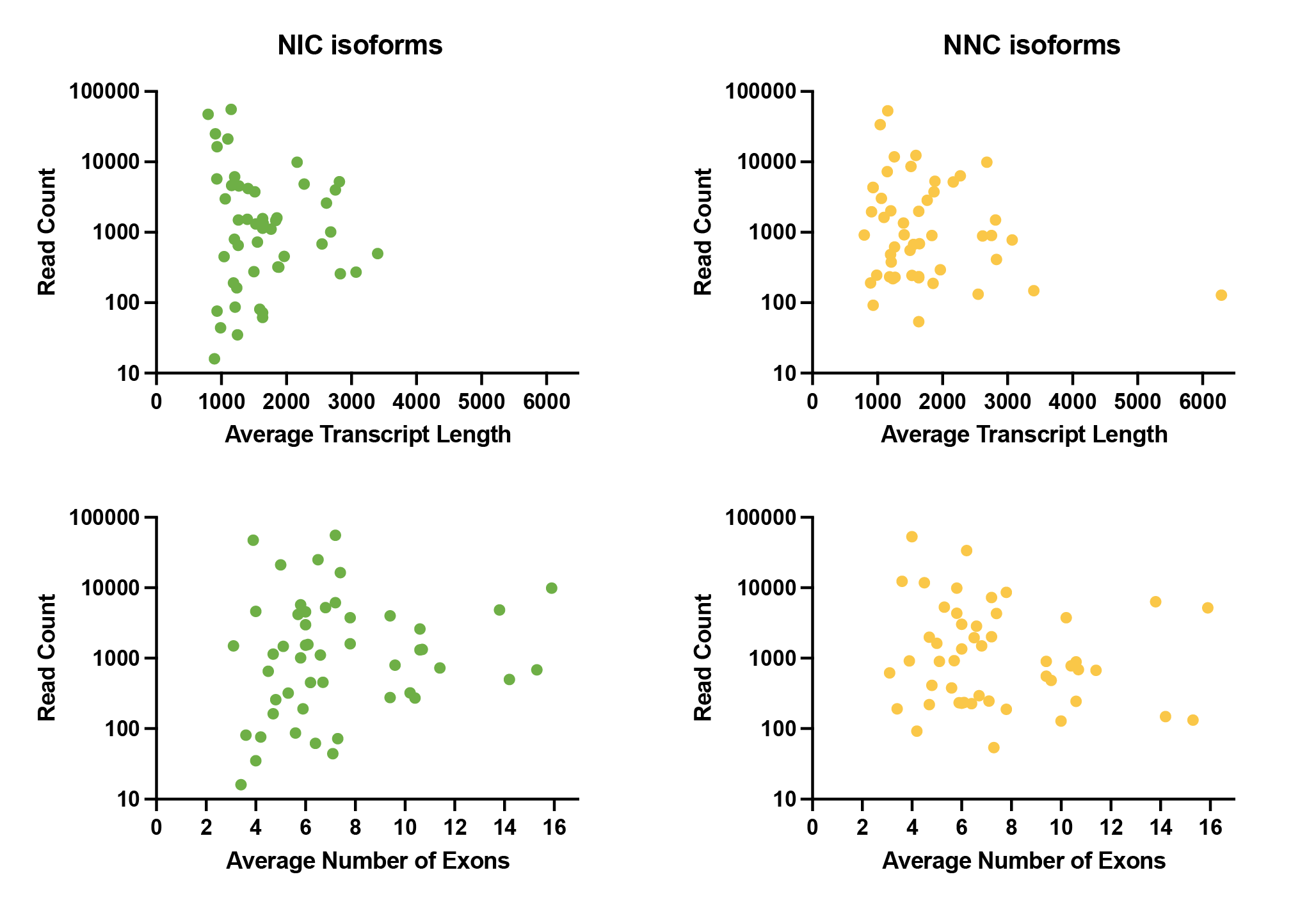

Supplement: Figure 5-2 — Novel isoform correlation with gene characteristics. Relationship between average transcript length and number of NIC (top left) or NNC (top right) reads. Each dot corresponds to a gene in our enrichment panel. Relationship between average number of exons in isoforms of a gene and the number of NIC (bottom left) or NNC (bottom right) reads. Each dot represents a gene. Download Figure 5-2, TIF file. [file eneuro-11-ENEURO.0296-24.2024-s010.tif]

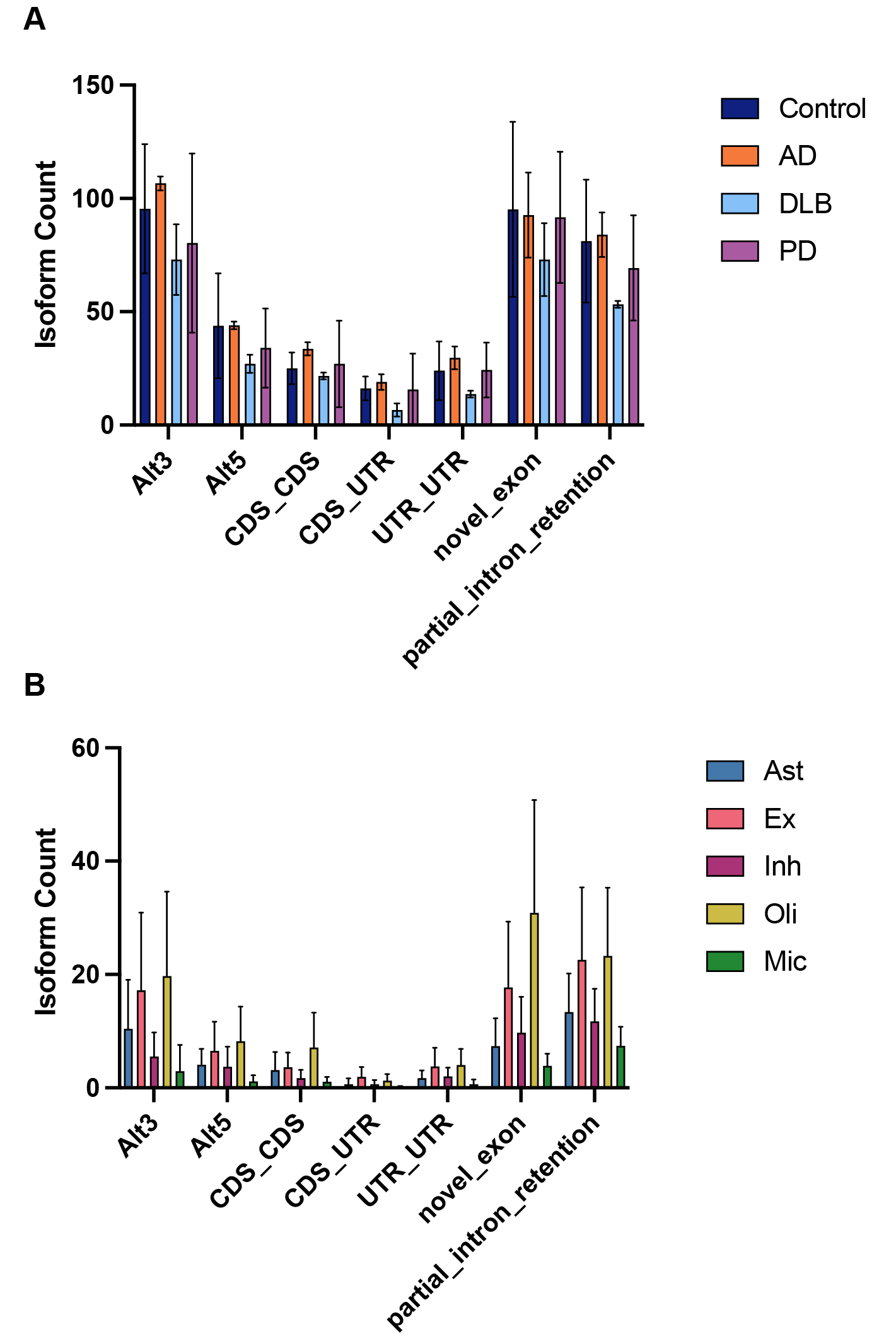

Supplement: Figure 5-3 — NNC features by disease group and cell type. (A) Number of NNC isoforms with a particular NNC feature per sample grouped by disease. Bar represents the mean across samples, and error bars represent standard deviation. (B) Number of NNC isoforms with a particular NNC feature per sample grouped by cell type. Bar represents the mean and error bars indicate standard deviation. Download Figure 5-3, TIF file. [file eneuro-11-ENEURO.0296-24.2024-s011.tif]

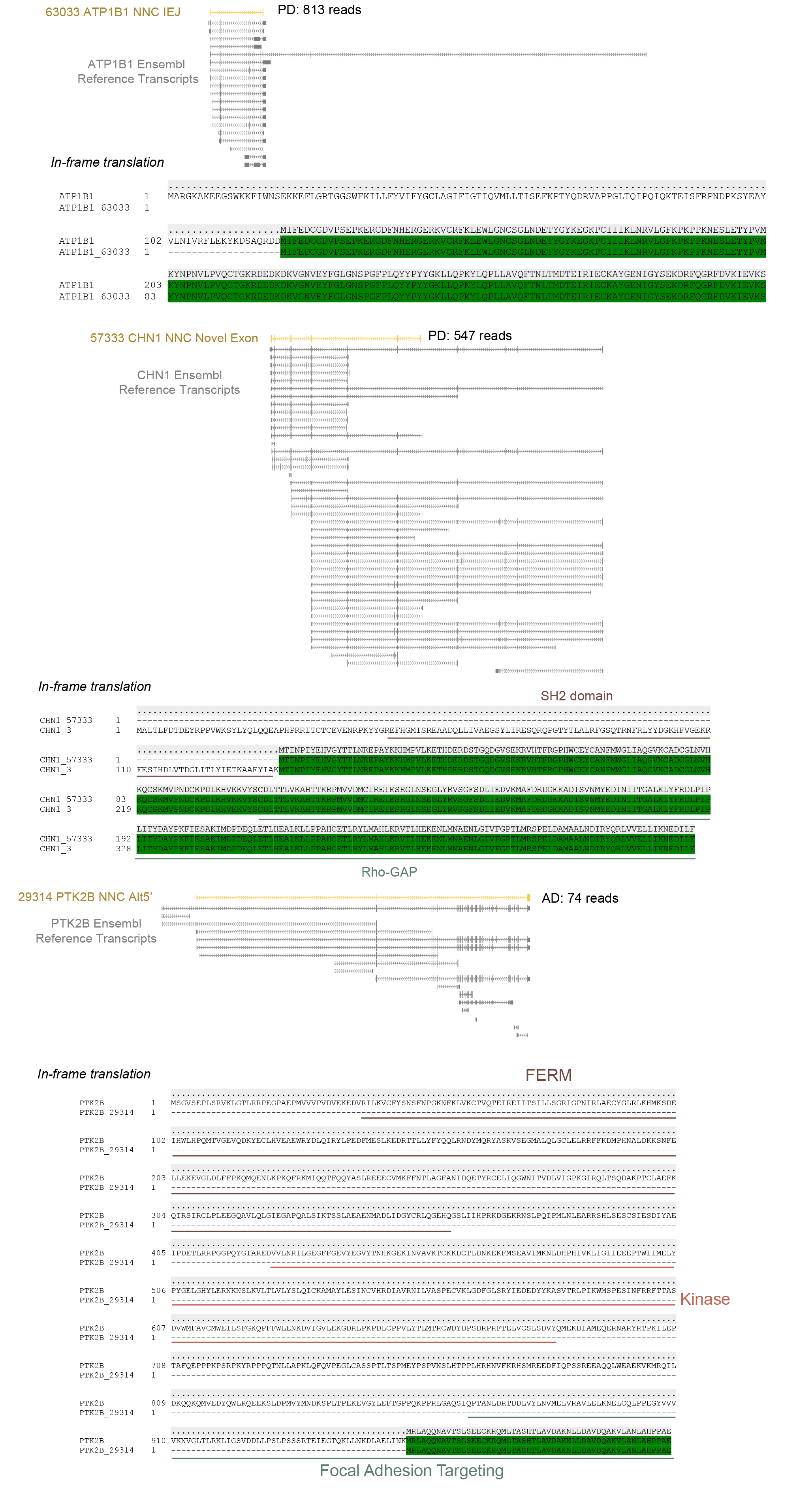

Supplement: Figure 6-1 — Select examples of NNC transcripts. The NNC transcript is shown in reference to Ensembl reference transcripts. In-frame translations of these NNC transcripts are aligned with the canonical protein isoform. Domains of full-length canonical proteins are highlighted to demonstrate loss of functional domains in the theoretically translated diseased isoform. Download Figure 6-1, TIF file. [file eneuro-11-ENEURO.0296-24.2024-s012.tif]
